# A comparison of diagnostic consistency for asthma-chronic obstructive pulmonary disease overlap and clinical characteristics study

**DOI:** 10.1186/s12890-019-1024-2

**Published:** 2019-12-18

**Authors:** Wenjing Ye, Xiaoming Li, Wen Gu, Xuejun Guo, Fengfeng Han, Song Liu

**Affiliations:** Department of Respiratory Medicine, Xinhua Hospital, Shanghai Jiaotong University School of Medicine, 1665, Kongjiang Road, Shanghai, 200092 China

**Keywords:** Diagnostic consistency, Asthma, Chronic obstructive pulmonary disease

## Abstract

**Background:**

The diagnostic criteria for asthma-chronic obstructive pulmonary disease overlap have not been unified. Different studies have used different criteria, and this has led to diagnostic inconsistencies.

**Methods:**

We collected data of patients who were older than 40 years and hospitalised because of chronic bronchial diseases. One hundred and seventy-one patients were included in this study. We compared seven different diagnostic criteria, examined their consistency, and analysed differences among groups classified with each set.

**Results:**

The prevalence of ACO ranged between 7.02 and 27.49% depending on the criteria applied. The patients who met the Soler-Cataluna et al. criteria also met the GesEPOC criteria. Rhee has proposed the strictest diagnostic criteria; hence, the number of patients who met these criteria was the smallest, and those patients also met the diagnostic criteria proposed by the other studies. We found that applying the different sets of criteria did not lead to the selection of the same population, while there were no statistical differences in age, disease duration, allergens, and inflammatory markers.

**Conclusions:**

The diagnostic criteria of ACO have not been unified, which hinders the design and progress of clinical studies that would investigate the ACO phenotypes and underlying mechanisms.

## Background

Chronic obstructive pulmonary disease (COPD) is preventable, common, and treatable and is characterised by persistent respiratory symptoms and airflow limitation [1]. Asthma is a common, chronic respiratory disease, characterised by variable symptoms of wheezing, shortness of breath, chest tightness and/or cough, and by variable expiratory airflow limitation [2]. In some patients, chronic asthma cannot be clearly distinguished from COPD using currently available tests and techniques, and in those patients, it is assumed that asthma and COPD coexist. Asthma-COPD overlap syndrome [3] has been coined to acknowledge that this represents an overlap of common disorders causing chronic airflow limitation rather than being a distinct syndrome [4]. To avoid the impression that this is a single disease, the term ACOS is no longer advised; the descriptive term asthma-COPD overlap (ACO) may be more appropriate [2]. The prevalence rates of ACO range from 15 to 55%, with variation depending on sex and age [5–7]. The wide range may be due to the different criteria used by different investigators. The prognosis of ACO is often worse than that of asthma or COPD alone [8], but the evidence for ACO treatment is very limited as few pharmacotherapy studies have examined this population. The different diagnostic criteria used in various regions and by different investigators might also limit the progress of ACO clinical studies. The diagnostic criteria of ACO have not been unified. Previous studies have used their own respective standards, so there was a lack of evaluation among the ACO patients screened by each standard. Our study compared the consistency among seven different sets of ACO diagnostic criteria proposed by previous studies to examine the clinical characteristics of patients screened by different standards, aiming to provide more clinical evidence for ACO diagnosis.

## Materials and methods

### Subjects

We collected data of patients who were older than 40 years and hospitalised because of chronic bronchial diseases (asthma or COPD) in Xinhua Hospital, from January 2017 to April 2018. The inclusion and exclusion criteria are shown in Table 1. One hundred and seventy-one patients were included in this study. The study was approved by the ethics committee of Xinhua Hospital, and informed consent was secured from the participating patients.

**Table 1:** Inclusion and exclusion criteria

Inclusion criteria	Exclusion criteria
1. Patients older than 40 years 2. Patients with chronic bronchial diseases (asthma or chronic obstructive pulmonary disease)	1. Patients with severe cardiac, hepatic, renal, or other organ dysfunction 2. Patients with other lung diseases (bronchiolitis obliterans, allergic bronchopulmonary aspergillosis, bronchiectasis, pulmonary embolism, tuberculosis, and others) 3. History of regular corticosteroid or other immunosuppressive agent use for other diseases 4. Patients who could not provide prior informed consent or delayed informed consent

### Data collection

Clinical data included general information (name, sex, age, age at onset, family history, smoking history), laboratory tests (routine blood test, eosinophilia in sputum, immunoglobulin E, arterial blood gas, allergen detection, and inflammatory factors), pulmonary function tests, disease condition in the past year, and medication use.High IgE meaned IgE > 100 IU/ml, high FeNO meaned FeNO > 25 ppb, elevated sputum eosinophil meaned eosinophil > 1.01% [9]. In order not to omit COPD patients, we equated exposures of noxious particles or gases over 10 years, such as tobacco smoke, air pollution, and occupational exposures to smoking history.

### Diagnostic criteria

Our study compared the consistency among seven different sets of ACO diagnostic criteria proposed by previous studies, including the GOLD in 2016 [10], Spanish COPD Guidelines (GesEPOC) [11], Soler-Cataluna et al. [12], Marsh et al. [6], Kauppi et al. [5], Louie et al. [13], and Rhee [14] (Table 2) .

**Table 2:** Seven sets of diagnostic criteria used by different investigators

Research	Diagnostic criteria
GOLD [9]	Five steps: • Diagnose chronic airways disease; • Syndromic diagnosis of asthma, COPD, and ACOS • Spirometry • Commence initial therapy • Specialised investigations if necessary
GesEPOC [10]	Major criteria: • Very positive bronchodilator test (increase in FEV1 > 15% and > 400 ml); • Eosinophilia in sputum; • Personal history of asthma; Minor criteria • High levels of total IgE; • Personal history of atopy; • Positive bronchodilator test on at least two occasions (increase of FEV1 > 12% and > 200 ml) 2 major criteria or 1 major and 2 minor criteria should be met
Soler-Cataluna et al. [11]	Major criteria: • Positive bronchodilator test (increase in FEV1 ≥ 15% and ≥ 400 ml); • Eosinophilia in sputum; • Personal history of asthma; Minor criteria: • High total IgE; • Personal history of atopy • Positive bronchodilator test (increase in FEV1 ≥ 12% and ≥ 200 ml) on two or more occasions. 2 major criteria and 2 minor criteria should be met
Marsh et al. [6]	Meet the definitions of COPD and meet more than one of the following: • Post-bronchodilation increase in FEV ≥ 15% • Peak flow variability ≥20% during 1 week of testing • Physician diagnosis of asthma in conjunction with current symptoms or inhaler use in the preceding 12 months.
Kauppi et al. [5]	Meet the definitions of COPD and meet more than one of the following: • Post bronchodilator increase in FEV1 of ≥12%; • Bronchodilator response of ≥15% or diurnal variation of ≥20% in PEF; • Moderate to severe bronchial hyperreactivity; • Decrease in FEV1 of ≥15% in the exercise test.
Louie et al. [12]	Major criteria: • A physician diagnosis of asthma and COPD in the same patient; • History or evidence of atopy; • Elevated total IgE; • Age 40 years or more, smoking > 10 pack-years; • Post bronchodilator FEV1 < 80% predicted and FEV1/FVC < 70%; Minor criteria: • Post bronchodilator FEV1 increase ≥15% or ≥ 12% and ≥ 200 ml increase in FEV1
Rhee [13]	At least one of the spirometric criteria for asthma (positive for bronchodilator response test; positive for provocation test) **and** at least two of the clinical criteria for asthma (history of asthma before the age of 40 years; elevated sputum eosinophil or FENO; history of allergic disease) **and** spirometric criterion for COPD (post FEV 1/FVC < 0.7) **and** clinical criterion for COPD (Smoking > 10 pack years)

### Statistical analysis

SPSS was used for statistical analysis. To compare differences among groups, analysis of variance, chi-square tests, and the Kruskal-Wallis test were used for parametric continuous, categorical, and nonparametric continuous variables, respectively. *P* < 0.05 was considered statistically significant. Diagnostic consistency was calculated by kappa testing, and kappa coefficients were assessed as follows: 0.01–0.40: slight agreement; 0.41–0.70: moderate agreement; 0.71–0.99: high agreement.

## Results

The data of 171 participants were analysed. The sample was 68.4% male and had a mean age of 67.5 years. In total, 115 cases (67.3%) had a history of smoking. Full details are shown in Table 3.

**Table 3:** General information of subjects

Variable	Count (%) or Mean (SD) or Median (IOR)	
Sex (Male)	117 (68.40%)	
Age,years	67.5 (10.19)	
Duration,years	6 (1–15)	
Smoking history	115 (67.30%)	
Smoking index (*n* = 115)	800 (400–1000)	
Exposures of noxious particles or gases	118(69.00%)	
EOS, * 10^6/L (*n* = 164)	100 (22–200)	
IgE,IU/ml	95.10 (35.40–402)	
Respiratory failure	34 (19.9%)	
IL-2,pg/ml (*n* = 162)	515 (397–707)	
IL-6,pg/ml (*n* = 162)	3.485 (2–11.05)	
IL-8,pg/ml (*n* = 162)	20.1 (11.75–44.58)	
TNF,pg/ml (*n* = 162)	12.45 (8.03–22.05)	
Positive results of allergens	48 (28.1%)	
Sum of positive allergen titers, IU/ml (*n* = 48)	0.95 (0.63–4.09)	
FEV1/FVC,%	58.14 (13.95)	
FEV1%pred,%	56.67 (23.43)	
RV/TLC,%	54.13 (9.11)	
FVC%pred,%	74.4 (62–91)	
FeNO,ppb (*n* = 170)	23 (12–41.25)	
Increased FEV1% after bronchodilation,%	7.2 (3.6–15.5)	
Increased FEV1 after bronchodilation,ml	90 (40–190)	
Very positive bronchodilator test	8(4.7%)	
Positive bronchodilator test	37(21.6%)	
Personal history of asthma	56(30.9%)	
High levels of total IgE	81(47.4%)	
Eosinophilia in sputum or high FeNO	72(42.4%)	
Health care utilisation in the past year	Hospitalised	6 (3.5%)
	Emergency	13 (7.6%)
	Out patient	152 (88.9)
Medication use	Oral or systemic CS	13 (7.6%)
	ICS + LABA+LAMA	37 (21.6%)
	ICS + LABA	55 (32.2%)
	LAMA	33 (19.3%)
	Others	33 (19.3%)

### Seven sets of ACO diagnostic criteria

**Fig. 1:**
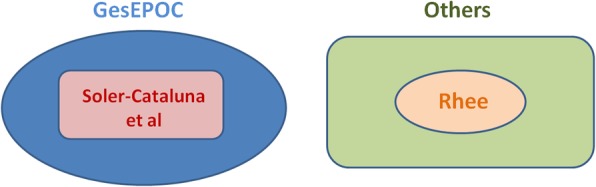
Seven sets of diagnostic criteria for asthma-chronic obstructive pulmonary disease overlap

The prevalence of ACO in chronic airway diseases ranged from 7.02 to 27.49% (Table 4). The patients who met the Soler-Cataluna et al. criteria also met the GesEPOC criteria. Rhee has proposed the strictest diagnostic criteria; hence, the number of patients who met these criteria was the smallest, and those patients also met the diagnostic criteria proposed by the other studies (Fig. 1).

**Table 4:** ACO prevalence based on the sets of diagnostic criteria

	GOLD [9]	GesEPOC [10]	Soler-Cataluna et al [11]	Marsh et al [6]	Kauppi et al [5]	Louie et al [12]	Rhee [13]
Met the criteria	43	47	37	46	31	22	12
Prevalence	25.15%	27.49%	21.64%	26.90%	18.13%	12.87%	7.02%

### Consistency comparison among the seven sets of ACO diagnostic criteria

The GesEPOC diagnostic criteria were most consistent with those proposed by Soler-Cataluna et al. (Kappa = 0.84, *P* < 0.0001). The Marsh et al. criteria were moderately consistent with the other sets of diagnostic criteria, while the criteria proposed by Rhee were poorly consistent with those proposed by the other studies (Kappa < 0.4 in all but one case) (Table 5).

**Table 5:** Consistency comparison among the seven sets of diagnostic criteria

	GOLD	GesEPOC	Soler-Cataluna	Marsh	Kauppi	Louie	Rhee
GOLD [9]	–	–	–	–	–	–	–
GesEPOC [10]	0.28**	–	–	–	–	–	–
Soler-Cataluna et al [11]	0.22**	0.84**	–	–	–	–	–
Marsh et al [6]	0.50**	0.42**	0.41**	–	–	–	–
Kauppi et al [5]	0.35**	0.15*	0.16*	0.59**	–	–	–
Louie et al [12]	0.24**	0.24**	0.17*	0.40**	0.58**	–	–
Rhee [13]	0.08	0.18*	0.16*	0.19**	0.35**	0.61**	–

**P < 0.0001

*P < 0.05

### Clinical characteristics of the patients with ACO based on the seven sets of diagnostic criteria

There were significant differences in sex, smoking history, and lung function (increase in forced expiratory volume in one second (FEV1) and FEV1% after bronchodilation) among the diagnostic criteria. However, we found no statistical differences in age, disease duration, positive results of allergens, and inflammatory markers (such as interleukin (IL)-2, IL-6, IL-8, tumour necrosis factor, and fractional exhaled nitric oxide) (Table 6).

**Table 6:** Clinical characteristics of ACO patients

	GOLD [9] (*n* = 43)	GesEPOC [10] (*n* = 47)	Soler-Cataluna et al [11] (*n* = 37)	Marsh et al [6] (*n* = 46)	Kauppi et al [5] (*n* = 31)	Louie et al [12] (*n* = 22)	Rhee [13] (*n* = 12)	*P*
Sex (Male)	26 (60.5%)	27 (57.4%)	21 (56.8%)	36 (78.3%)	29 (93.5%)	21 (95.5%)	10 (71.4%)	*P* < 0.0001
Age,years	65.72 (9.00)	65.06 (9.79)	64.41 (9.74)	65.46 (9.77)	65.10 (10.98)	65.82 (9.37)	62.58 (10.55)	*P* > 0.05
Duration,years	10 (1–20)	10 (5–40)	10 (5–40)	5.5 (1.75–15)	4 (1–12)	4 (1–12)	5.5 (1.75–14.25)	*P* > 0.05
Exposures of noxious particles or gases	28 (65.1%)	29 (61.7%)	21 (56.8%)	39 (84.7%)	29 (93.5%)	22 (100%)	12 (100%)	*P* < 0.0001
EOS,* 10^6/L	44 (22–200)	100 (22–286)	100 (22–260)	100 (22–205)	100 (22–200)	200 (22–273)	177 (22–215)	*P* > 0.05
IgE,IU/ml	107 (40–427)	309 (49.3–647)	384 (103.1–727.5)	153 (47.28–470.75)	138 (34.4–402)	312.5 (105.75–706.25)	395 (179.75–996)	*P* > 0.05
IL-2,pg/ml	539.54 (252.81)	465.7 (211.6)	447.57 (198.03)	533.16 (276.04)	547.38 (288.53)	534.30 (242.03)	546.82 (268.23)	*P* > 0.05
IL-6,pg/ml	3.26 (2–11.6)	2.7 (2–5.86)	2.86 (2–6.33)	3.45 (2.15–11.6)	5.86 (2.63–14.35)	3.33 (2.33–11.83)	3.14 (2.31–7.63)	*P* > 0.05
IL-8,pg/ml	21.3 (11.2–66.6)	17.3 (10.23–32.55)	16.1 (10.3–33.1)	19 (11.9–49.1)	21.7 (12.5–31.55)	22.2 (10.08–32.63)	19.9 (9.08–27.7)	*P* > 0.05
TNF,pg/ml	18 (6.92–23.1)	12.7 (6.63–22.43)	13 (6.66–26.1)	17.2 (8.88–27.5)	12.3 (8.17–23.7)	12.5 (8.12–19.85)	10.2 (6.62–16.4)	*P* > 0.05
Positive results of allergens	21 (53.8%)	24 (61.5%)	20 (69%)	22 (51.2%)	11 (42.3%)	8 (47.1%)	6 (66.7%)	*P* > 0.05
FEE1/FVC,%	59.35 (13.90)	60.95 (14.71)	59.81 (14.41)	58.32 (13.70)	54.57 (11.44)	56.66 (9.97)	59.56 (10.93)	*P* > 0.05
FEV1pred,%	58.95 (22.97)	59.78 (25.15)	58.51 (25.08)	56.19 (23.46)	50.45 (18.62)	54.24 (17.78)	59.59 (20.02)	*P* > 0.05
FVCpred,%	77 (19.98)	75.478 (20.08)	75.318 (20.798)	73.428 (19.90)	69.94 (18.10)	72.46 (16.43)	76.13 (18.94)	*P* > 0.05
RV/TLC,%	54.33 (8.84)	53.34 (7.61)	53.72 (7.71)	53.12 (8.04)	54.65 (7.70)	54.77 (7.52)	54.91 (7.84)	*P* > 0.05
FeNO,ppb	27.91 (26.16)	42.36 (41.52)	41.14 (32.01)	33.61 (31.11)	28.19 (19.83)	33.86 (42.30)	45.92 (54.47)	*P* > 0.05
Increased FEV1% after bronchodilation,%	13.90 (14.25)	13.62 (13.67)	14.85 (15.00)	17.10 (14.94)	25.14 (12.66)	20.69 (12.83)	20.2 2(14.10)	*P* < 0.05
Increased FEV1 after bronchoditation,ml	180 (173.95)	169.79 (154.35)	180.27 (170.25)	220.22 (169.20)	330 (116.56)	305.45 (134.23)	325.83 (146.38)	*P* < 0.0001

## Discussion

Most previous studies of airways diseases have excluded patients with ACO [15, 16]. We collected data of 171 patients who were older than 40 years and had chronic bronchial diseases. After comparing seven sets of diagnostic criteria, examining their consistency, and analysing differences among groups, we found that the different sets did not lead to the selection of the same population.

Based on the lung function test definition of ACO alone, many patients with asthma or COPD could be considered to have ACO. Thus, a narrower and more accurate definition of ACO is needed in clinical practice. Some experts have suggested a definition of ACO based on both lung function and on clinical features [12]. However, a specific definition for ACO cannot be confirmed until more evidence becomes available regarding its clinical phenotypes and underlying mechanisms.

According to GOLD [10], clinicians should diagnose chronic airways disease first, estimate the syndromic diagnosis of asthma, COPD, and ACO; perform spirometry testing, and then commence therapy to estimate the therapeutic effect and confirm the diagnosis; additional specialised investigations should be performed if necessary. There is no specific numerical standard in GOLD, and it relies more on clinical symptoms and clinical judgment. Therefore, the GOLD are more descriptive of ACO. GOLD highlights the significance that the therapeutic effect has for diagnosis and does not rely on a single medical record, while the therapeutic effect was rarely mentioned in the other studies [5, 6, 11–14]. This renders this method more comprehensive, but it may also require a greater degree of subjective clinician input, leading to diagnostic inconsistencies and hence, it cannot be readily established as a standard.

The GesEPOC [11] and Soler-Cataluna et al. [12] criteria shared the most similarities. The GesEPOC criteria are relatively broad, and thus the patients who met the Soler-Cataluna et al. criteria also met the GesEPOC criteria. The common feature of these two sets of criteria are the high requirements regarding the bronchodilator test. Both sets of criteria require a very positive bronchodilator test (major criterion; increase in FEV1 ≥ 15% and ≥ 400 ml) and positive bronchodilator tests on two or more occasions (minor criterion; increase in FEV1 ≥ 12% and ≥ 200 ml). While the criteria proposed by the other studies mostly require one positive bronchodilator test. This might be the reason why these two sets had poor consistency with the criteria proposed by the other studies.

The Marsh et al. [6] and Kauppi et al. [5] diagnostic criteria are both based on a definitive diagnosis of COPD first, and then, to be diagnosed with ACO, the patients should have certain asthma characteristics. The asthma characteristics included in the Kauppi et al. study were only focused on lung function, while Marsh et al. also included physician diagnosis of asthma and inhaler use. Therefore, the Marsh et al. assessment is more comprehensive, and this might explain why the Marsh et al. diagnostic criteria, compared to the Kauppi et al. criteria, were generally more consistent with those proposed by the other studies.

The Rhee diagnostic criteria are the most stringent, stating that the patients should meet the spirometric and clinical criteria for asthma and the spirometric and clinical criteria for COPD [14]; the criteria proposed by the other studies only required patients to meet some of these to receive an ACO diagnosis. Consequently, the fewest patients were diagnosed with ACO based on the Rhee criteria, which led to the worst consistency between this set and the other sets of criteria. However, the patients who meet the Rhee criteria might be considered to have true ACO. Without a firm definition of ACO, it is not possible to perform high-quality clinical trials. Thus, Rhee contested that a narrow definition of ACO, which includes both asthma and COPD diagnoses, was needed.

In order to delineate the clinical characteristics of the patients selected by each diagnostic method, we statistically compared the groups. It was found that there was a significant difference in lung function, especially in increase in FEV1% and in FEV1. This might be in line with the GesEPOC [11] and Soler-Cataluna et al. [12] criteria, which require a very positive bronchodilator test. In addition, sex and smoking history were also statistically different among the groups. Some diagnostic criteria used smoking history as a major criterion while others as a minor criterion, and most of the smokers in the sample were male, which may have led to the significant differences. No differences were found in inflammatory factors and other biomarkers. Differences in biomarkers may be related to underlying mechanisms and phenotypes [17–19]. The diagnostic criteria examined here can only be used to diagnose ACO, and at present, there are no methods to diagnose the ACO phenotypes; further research is needed to this end [2].

The small sample and our single-center design limited the results of our study. We enrolled only patients with hospitalization. Many stable chronic airway disease patients without history of hospitalization were not included. This is indeed one of the shortcomings of our research. At the beginning of the study design, we also considered to include stable chronic airway disease patients of outpatient department into our study. However, most of those patients have incomplete examination data, and it is difficult to be included in the statistics. So finally we decided to enroll only patients with hospitalization. Additionally, our study was cross sectional and it did not involve follow-up observation of the clinical characteristics and treatment effects in our patients. Our team plans to follow up the patients in a prospective study, aiming to advance the search for the most appropriate diagnostic criteria for ACO and its phenotypes.

## Conclusions

The diagnostic criteria of ACO have not been unified, and the diagnostic methods used in different studies lead to diagnostic inconsistencies. Furthermore, these methods cannot be used to diagnose the ACO phenotypes and to study the underlying mechanisms. As a future research direction, our team plans to follow up on the clinical characteristics and treatment effects in these patients in a prospective study to propose some potential solutions.

## Data Availability

Please contact Wenjing Ye.

## Abbreviations

ACOS: Asthma-COPD overlap syndrome
COPD: Chronic obstructive pulmonary disease
FEV1: Forced expiratory volume in one second
IL: Interleukin
